# Trait emotional intelligence and vocational outcome expectations among Turkish preservice teachers: serial mediating role of hope and career adaptability

**DOI:** 10.3389/fpsyg.2026.1770725

**Published:** 2026-04-20

**Authors:** Hazel Duru, Filiz Gültekin

**Affiliations:** Department of Psychological Counseling and Guidance, Faculty of Education, Bursa Uludağ University, Bursa, Türkiye

**Keywords:** career adaptability, hope, preservice teacher, trait emotional intelligence, vocational outcome expectations

## Abstract

**Introduction:**

This cross-sectional study investigated the sequential mediating effect of hope and career adaptability on the relationship between trait emotional intelligence and vocational outcome expectations among preservice teachers.

**Methods:**

Data were collected from 311 preservice teachers (239 female, 72 male) across various teaching disciplines. The SPSS Amos software package was used for data analysis.

**Results:**

The findings indicate that trait emotional intelligence is significantly associated with vocational outcome expectations. Crucially, the results demonstrate that both hope and career adaptability collectively mediate this relationship through a sequential mediation effect.

**Discussion:**

These results reveal that vocational outcome expectations are also shaped by emotional and motivational resources, thereby contributing to the existing literature on the professional development processes of preservice teachers.

## 1 Introduction

Vocational choice is the result of a decision, and behind this decision lie the individuals’ expectations from that profession and the outcomes they seek to obtain (Lent et al., 1994). Studies focused on an individual’s vocational development aim to assist the person in selecting an occupation that is most suitable for their self-concept and expectations (Super, 1990). Vocational outcome expectations (VOE) are a more specific manifestation of general outcome expectations. VOE represents beliefs about the long-term achievement results to be attained through educational/vocational decision-making processes (Betz and Voyten, 1997). Integrated with specific occupational values such as earnings and prestige (Lent et al., 1994), VOE also refers to individuals’ beliefs regarding the likelihood of achieving these vocational values through their chosen profession (Işık, 2013).

Outcome expectation is one of the three core constructs (self-efficacy, outcome expectations, and personal goals) of Social Cognitive Career Theory (SCCT) for explaining career development. Self-efficacy refers to an individual’s belief in their capacity to complete a particular task successfully. Personal goals reflect the strength of an individual’s intention to engage in specific behaviors. Outcome expectations encompass predictions regarding the potential physical, social, and self-evaluative consequences of such actions (Buthelezi et al., 2009). These personal cognitive factors, particularly self-efficacy and outcome expectations, play a critical role in shaping career interests; these interests guide career choices and influence performance outcomes (Lent et al., 1989). Literature shows that VOE is related to career concepts such as career-seeking behaviors (Ireland, 2020; Klein et al., 2021; Patton et al., 2004), entrepreneurial intention (Santos and Liguori, 2020), goal commitment (Wu et al., 2024), job satisfaction (Lee et al., 2020), and career goals (Chan, 2020). While VOE is primarily associated with professional achievement and values, studies have shown that this construct can indirectly influence broader life outcomes such as life satisfaction and psychological wellbeing (Yılmaz et al., 2020; Cadenas et al., 2021). The relationship between VOE and career success and life outcomes highlights the importance of examining this construct, particularly among young adults. Given the critical role of teachers in society, understanding the factors that shape professional outcome expectations (VOE) in prospective teachers is considered crucial.

Teaching is a profession that plays a critical role in ensuring the academic and personal development of individuals at different developmental stages. Therefore, it requires many competencies. In Turkey, teachers are expected to communicate effectively with all stakeholders in the education process, continuously evaluate their development, and improve themselves professionally (The Ministry of National Education [MoNE], 2017). Teaching is a challenging profession for many reasons. Research shows that professional burnout is high among teachers (Avcı and Seferoğlu, 2011; Kıral and Diri, 2016; Erkul and Dalgıç, 2014). Like the teaching profession itself, the preparation process also involves many problems. Many prospective teachers experience difficulties during the professional preparation process and do not feel ready for the realities of the profession (Karakuş, 2011). Problems such as the inadequacy of teacher training programs in terms of implementation, the mismatch between the curriculum and school realities, and the lack of recognition of competencies weaken the motivation and effectiveness of prospective teachers (Tuli and Oljira, 2020), increase feelings of inadequacy, and lead to a lack of confidence in their teaching skills (Karakuş, 2011). Employment is also an important factor influencing prospective teachers’ expectations. In Turkey, 572,019 teachers applied for the exam to work in public schools in 2023 (ÖSYM- Measuring, Selection and Placement Center, 2023). Education faculties are admitting more students than needed. For example, the number of places in sports teaching departments in the last 5 years is 16,954. The number of teachers employed in public schools is 3,299 (Taflı and Yetiş, 2025). In short, expectations regarding a profession can be influenced by the individual’s characteristics, the profession itself, and the society in which the individual lives. Research shows that micro-level factors (interacting with the environment, maintaining control over situations, and achieving significant results) are as important as macro-level factors (economic conditions and policies) in individuals’ career development (Savickas et al., 2009; Duffy et al., 2016). In this context, since coping with systemic uncertainties and employment anxiety requires substantial emotion regulation, personal resources are critical for professional outcomes (Atitsogbe et al., 2019). Consequently, trait emotional intelligence (EI) emerges as a fundamental can be considered a personal trait linked to an individual’s belief in professional success.

Salovey et al. (2000) suggest that an individual’s life success depends on how they manage their emotions and their ability to process relevant information. Research shows that trait EI is positively correlated with employee commitment (George et al., 2022; Kumari and Priya, 2015), career-building skills (Kırıkkanat, 2023), career decision-making self-efficacy (Jiang, 2014), and career success (Sharma and Tiwari, 2023; Çiçeklioğlu, 2024); and negatively correlated with career decision-making difficulties (Ran et al., 2022). Accordingly, trait EI can be considered an important variable for career success. It is assumed that trait EI provides individuals with a sense of control in performing career-related tasks and positive beliefs about achieving their goals (Young et al., 1996). Because individuals with high trait EI can effectively regulate negative emotions when facing systemic barriers, they are better equipped to sustain these positive beliefs and generate alternative pathways to their goals. This cognitive and motivational process directly evokes hope, another essential personal resource. Hope is a cognitive process based on an individual’s belief that they can achieve their desired goals (Snyder et al., 1991b). Hope is not merely a wish, but a desire to achieve goals and a belief in the ability to plan for them. When faced with challenges, hopeful individuals are more inclined to generate alternative solutions and move toward their goals (Snyder, 2005; Snyder et al., 1998). As an internal and cognitive process that enables the formation of positive expectations for the future, hope can be a variable related to VOE and, through emotion regulation/control mechanisms, to emotional intelligence. Understanding and managing emotions, the desire to achieve goals, and the ability to move toward them can enable an individual to better cope with the adversities or changes they may encounter on their career journey. This goal-directed planning and proactive adjustment to challenging professional environments naturally foster career adaptability. Therefore, career adaptability, which refers to an individual’s readiness and capacity to cope with adverse situations and adapt to changes in their professional life (Savickas, 1997), serves as a crucial bridge in this context. This skill may be positively correlated with individuals’ beliefs in achieving greater professional success in the future. In summary, VOE is a critical concept for young adults’ careers, and it is theoretically proposed that a sequential mechanism exists among these personal and motivational variables: prospective teachers’ trait EI fosters higher levels of hope, which in turn strengthens their career adaptability in the face of employment and professional challenges, ultimately enhancing their VOE. Based on this theoretical articulation, this study differs from previous studies by addressing tEI, hope, and career adaptability within a holistic framework to understand prospective teachers’ professional outcome expectations. It aims to determine the role of personal resources, emotional and motivational processes, such as tEI, hope, and career adaptability, in explaining professional outcome expectations. These personal resources are characteristics that can enable individuals to mobilize their potential in career development. It offers insights into the role of tEI in supporting VOE through the serial mediating effect of hope and career adaptability. Studies examining the direct or indirect effects of these variables on VOE, especially those targeting prospective teachers, are limited. This study, which combines positive psychology and career development approaches, is expected to provide theoretical support for the role of personal factors in career development research. Işık (2010) states that VOE is a variable construct and an important factor in the development of an individual’s self-perceptions, attitudes, goals, and interests. In this context, the study aims to contribute to career development practices by identifying variables associated with positive career-outcome expectations among prospective teachers.

## 2 Literature review

### 2.1 Trait emotional intelligence and vocational outcome expectations

Emotional intelligence refers to an individual’s ability to perceive themselves and others, understand, and manage their emotions (Koman and Wolff, 2008). Emotional intelligence, as an ability, is a distinct type of intelligence that involves reasoning about emotions (Mayer et al., 2008). Emotional intelligence as a trait is a set of emotion-related dispositions determined by higher-order personality dimensions (Petrides et al., 2007). Trait EI is concerned with individuals’ perceptions of their emotional world (Petrides et al., 2016). Individuals can only access direct information about their emotional capabilities related to their internal processes. Based on this, trait EI can be used as a functional definition that describes the subjectivity of emotional experiences (Petrides, 2010). Trait EI is highly functional compared to other emotional intelligence approaches because it acknowledges that an individual’s emotional experiences are subjective, is not dependent on specific tests, and is empirically supported (Robinson and Clore, 2002; Petrides, 2010).

Trait EI provides motivation and self-regulation skills for achieving career goals and planning for career adaptability (Coetzee and Harry, 2014; Parmentier et al., 2019). Pirsoul et al. (2023) investigated emotional intelligence as a regulatory resource predicting career-related outcomes in their study. Studies have shown that emotional intelligence is related to employee wellbeing (Karimi et al., 2021) and is a significant predictor of positive happiness and optimism (Tejada-Gallardo et al., 2022), as well as career optimism (Kaya and Sart, 2020). Although some studies have examined emotional intelligence in relation to career development variables, no research has investigated it within the context of VOE. In a study conducted by Duman and Taş (2021) with education faculty students at a university in Türkiye, participants stated that they viewed teaching as an exciting and enjoyable profession, where they loved learning and teaching, and that it touched and inspired the lives of children. Mancini et al. (2022) found, in their study with teachers, that emotional intelligence was a predictor of job-related emotional exhaustion and a lack of personal accomplishment. Kostić-Bobanović (2020) also found a relationship between emotional intelligence and teacher self-efficacy. In this context, trait EI may be a variable related to positive outcome expectations for preservice teachers. Consequently, we propose the following hypothesis:

*H1*: Trait EI is significantly and positively associated with VOE among preservice teachers.

### 2.2 The mediating role of hope in the relationship between trait emotional intelligence and vocational outcome expectations

Individuals with high levels of emotional intelligence can cope with stressful situations more effectively and better tolerate the negative effects of pressure while performing (Petrides et al., 2004). One of the emotions associated with emotional intelligence is hope. Research indicates that emotional intelligence is positively related to hope (Sarıçam et al., 2015) and negatively related to hopelessness (Yılmaz, 2019), suggesting that emotional intelligence is a significant predictor of hope (Batool et al., 2014; Mousa and Kamel, 2017). Hope is an emotion associated with an individual’s belief that life is worth living (Zournazi, 2004). It enables the individual to cope effectively with the difficulties they face (Sütütemiz et al., 2009). In Snyder’s model, one of the leading hope models in the literature, hope is a positive motivation based on a successful emotion that emerges interactively. It has two dimensions: goal-directed activity and plans to reach the goal (Snyder et al., 1991a). Hope is a cognitive process that involves an individual’s perceptions of their ability to achieve their goals (Snyder et al., 1991b). It comprises three components: goals, pathways thinking, and agency thinking (Snyder et al., 2002). An individual’s goals must have a specific value, be attainable, and involve a degree of uncertainty (Snyder, 2002, 2005; Snyder et al., 2002). Pathways thinking refers to the ability to create options and develop plans that can be used to achieve desired goals (Cheavens et al., 2006; Snyder et al., 2002). In this way, individuals can generate new solutions and achieve their goals through internal suggestions when they encounter various problems while pursuing their objectives (Snyder, 2005; Snyder et al., 1998). Agency thinking is a motivational element that provides individuals with the power to generate and select solutions when faced with problems (Snyder, 2002). Hope is essential for positive career development (Hirschi, 2014; Niles, 2011). For example, the solutions individuals propose for problems encountered in their work environments vary according to their levels of hope (Peterson and Byron, 2008). Hope predicts career adaptability (Büyükgöze-Kavas, 2016; Wilkins et al., 2014). Garcia et al. (2019) revealed that VOE increases as adolescents’ levels of hope increase. Duff and Chan (2014) stated that high levels of hopelessness lead individuals to evaluate themselves as failures in their careers. Factors such as confidence, hope, optimism, and resilience have a positive influence on many employee behaviors (Yalçın, 2017). Luthans et al. (2004) suggested that focusing on malleable concepts, such as confidence, hope, optimism, and resilience, in the workplace can enhance both individual and organizational performance. In this context, hope may be related to preservice teachers’ belief that their chosen profession can provide them with the life they desire. Accordingly, we propose the following hypothesis:

*H2*: Hope mediates the relationship between trait EI and VOE.

### 2.3 The mediating role of career adaptability in the relationship between trait emotional intelligence and vocational outcome expectations

Individuals need to continuously develop themselves to adapt to the changing world of work (Maggiori et al., 2013; Nota et al., 2014). Career adaptability refers to an individual’s ability to cope with tasks and unforeseen changes in their professional life (Savickas, 1997). Career adaptability consists of four dimensions (Savickas and Porfeli, 2012). Career concern refers to an individual’s awareness of their competencies and planning for their responsibilities (Savickas, 1991). Career control refers to the self-regulation skills that enable individuals to take responsibility for making choices about their future and their profession (Brown and Brooks, 1991). Career curiosity refers to an individual’s appraisal of educational opportunities and potential options related to their profession (Blustein, 1992). Career confidence refers to an individual’s ability to solve their problems (Hartung et al., 2008). In summary, this concept refers to an individual’s need to establish a career and take the necessary steps to achieve it. Trait EI significantly contributes to career adaptability and is a key predictor of it (Coetzee and Harry, 2014; Hamzah et al., 2021). Merino-Tejedor et al. (2024) state that trait EI has a positive spillover effect on vocational identity, strengthening it through career construction. Parmentier et al. (2019) specified that emotional intelligence is a general adaptation readiness factor upon which individuals can rely to develop and activate career adaptation resources. There is extensive literature regarding the relationship between emotional intelligence and career adaptability (Ahmed et al., 2022; Cinel and Oğan, 2021; Ginting, 2022; Harry and Malepane, 2021; Kaya and Sart, 2020; Merino-Tejedor et al., 2018; Pirsoul et al., 2023; Pong and Leung, 2023). Vashisht et al. (2023) reported a meta-analytic correlation between emotional intelligence and career adaptability.

Udayar et al. (2018) found that career adaptability mediates the relationship between emotional intelligence and perceived employability, as well as career decision-making difficulties. Studies have shown that career adaptability is negatively related to career stress (Duru and Gültekin, 2019) and positively related to career optimism (McLennan et al., 2017). Career indecision (Yiğit, 2018), as well as hope and optimism (Wilkins et al., 2014), predict career adaptability. Career adaptability is indirectly associated with life satisfaction through hope (Di Maggio et al., 2022). Cabras and Mondo (2018) concluded that university students who are confident in their career adaptability are more satisfied with life because they are more certain about their future expectations. However, adaptability is crucial for preservice teachers. Life and career skills focus on flexibility, adaptability, initiative, and self-direction (Partnership for 21st Century Skills, 2019). In other words, adaptability is one of the life and career skills. Primarily, it appears crucial for teachers to adapt to innovations, their environment, and their profession in order to increase productivity and make a positive impact on the students they interact with (Aygün et al., 2016). Very few studies examine career adaptability in the context of VOE. When viewed as a career-related coping resource, career adaptability may be related to trait EI and VOE. Therefore, we hypothesize that (H3) career adaptability is a mediator between trait EI and VOE.

### 2.4 Serial mediating role of hope and career adaptability in the relationship between trait emotional intelligence and vocational outcome expectations

This study investigates the relationship between trait EI, hope, and career adaptability with VOE among preservice teachers. Preservice teachers face administrative, instructional, procedural, psychological, and professional challenges (Pena, 2018). Preservice teachers often feel inadequate when working with culturally and linguistically diverse students (Piwowarski, 2024) and when coping with crises and bullying (Salo and Kajamies, 2024). VOE is individuals’ beliefs about the outcomes they will achieve in their profession. The positive nature of these beliefs can have a profoundly positive influence on an individual’s career development. Identifying the variables associated with an individual’s expectations regarding their profession can support relevant interventions.

Previous research has shown that emotional intelligence is associated with competence and effectiveness in teachers (Laput and Oropa, 2025; Kostić-Bobanović, 2020; Rahman et al., 2024). These results suggest that emotional intelligence may be a variable that supports professional competence and positive expectations regarding professional life. Specifically, emotional intelligence can help individuals maintain positive expectations about life. In other words, it can increase their level of hope. The current literature shows that emotional intelligence is associated with hope (Batool et al., 2014; Mousa and Kamel, 2017; Sarıçam et al., 2015; Yılmaz, 2019) and that hope is associated with career adaptability (Büyükgöze-Kavas, 2016; Wilkins et al., 2014). The proposed serial mediation model (Trait EI—Hope—Career Adaptability—VOE) is grounded in Snyder’s (2002) Hope Theory and Career Construction Theory (CCT; Savickas, 2013). CCT posits a sequential progression where adaptivity (personality traits) leads to adaptability resources (psychological capital), which in turn mobilize adapting responses (behaviors), ultimately resulting in adaptation results (outcomes). Within this sequential framework, Trait EI represents the initial adaptivity as a stable personality trait reflecting emotion regulation and competence. According to positive psychology frameworks, the ability to effectively manage emotions provides the cognitive and emotional foundation necessary for developing psychological resources. Specifically, Trait EI enables individuals to maintain a positive sense of agency and envision alternative pathways, which are the fundamental components of hope (Snyder, 2002). Therefore, Trait EI theoretically precedes hope and fosters it as a psychological resource. Subsequently, hope functions as an adaptability resource that drives proactive career behaviors. Individuals with high levels of hope are intrinsically motivated (agency) and capable of generating alternative solutions when faced with obstacles. According to CCT, these psychological resources activate adapting responses, conceptualized in this study as career adaptability. Hope empowers individuals to explore and plan complex professional processes and navigate career transitions with confidence.

Finally, when individuals effectively adapt to professional changes and successfully cope with career challenges, they develop more positive and realistic vocational outcome expectations (VOE). Consequently, it can be concluded that a foundational emotional trait (Trait EI) builds a motivational resource (hope), which subsequently activates career-related behaviors (career adaptability) and ultimately shapes positive vocational expectations (VOE). Accordingly, we hypothesize that (H4) hope and career adaptability are sequential mediators in the relationship between trait EI and VOE.

## 3 Materials and methods

### 3.1 Participants

The study group for the research consisted of 311 participants (239 female, 72 male) who were preservice teachers in Turkey. The age range of the research group is 18–48, with an average age of 21.2 (SD = 2.91). The researchers administered the scales to the study participants. Before applying the scales, information about the research was provided, and the participants’ consent was obtained. Before filling out the scales, participants were asked to read the instructions for each scale. The research was conducted in accordance with the ethical guidelines and received approval from the University’s Research and Publication Ethics Committee.

### 3.2 Measures

#### 3.2.1 Vocational outcome expectation scale

The scale, consisting of 12 items and one dimension, was developed by McWhirter et al. (2000) and adapted into Turkish by Işık (2010). Higher scores on the scale indicate higher vocational outcome expectations. The Cronbach alpha internal consistency coefficient of the scale, which was scored as a four-point Likert scale (e.g., “My career plan will lead to a result that will satisfy me.”), A validity coefficient of 0.87 was calculated. Valid fit index values were obtained in the confirmatory factor analysis of the scale. As a result of confirmatory factor analysis, the fit index values for the one-dimensional model were determined to be (GFI = 0.93, AGFI = 0.84, CFI = 0.92, NFI = 0.90, and RMSEA = 0.05) (Işık, 2010). The Cronbach alpha reliability coefficient calculated in this study is 0.85.

#### 3.2.2 Career adaptability scale

The scale, which was developed by Savickas and Porfeli (2012) and adapted into Turkish by Kanten (2012), consists of 19 items and four sub-dimensions: concern, control, curiosity, and confidence (e.g., “I research options before making a decision.”). Confirmatory factor analysis of the scale found the goodness of fit values (RMSEA = 0.07, SD = 144, and χ^2^ = 517.62), and the goodness of fit values were found to be higher than 0.90. The increase in the scores obtained from the sub-dimensions indicates that the perception of ability toward the relevant sub-dimension is high. As a result of the reliability analysis conducted by Kanten (2012), the Cronbach alpha reliability coefficients for the sub-dimensions of the scale are as follows: concern 0.61; control 0.77; curiosity 0.79; and confidence 0.81. In this study, the Cronbach alpha reliability coefficients for the sub-dimensions of the scale, concern 0.84; control 0.78; curiosity 0.76; and confidence 0.77 were calculated.

#### 3.2.3 Trait emotional intelligence questionnaire-short form

The scale was developed by Petrides and Furnham (2001) and adapted by Deniz et al. (2013). The scale consists of 20 items, each scored on a seven-point Likert scale (e.g., “I often have difficulty regulating my emotions.”). The scale has four subsections: wellbeing, emotionality, sociability, and self-control. High scores obtained from the scale indicate high emotional intelligence adequacy. For the whole scale, the reliability was calculated as 0.81 and the test-retest as 0.86. According to the confirmatory factor analysis, model fit indices were calculated as (χ^2^/df = 2.46, GFI = 0.95, AGFI = 0.92, CFI = 0.91, RMSEA = 0.056, and SRMR = 0.060). The alpha reliability coefficient calculated in this study is 0.81.

#### 3.2.4 Dispositional hope scale

It was developed by Snyder et al. (1991a) and adapted into Turkish by Tarhan and Bacanlı (2015). The scale consists of 12 items and two sub-dimensions: thinking of alternative pathways and agency thinking. Each item is evaluated using an eight-point Likert-type rating scale (e.g., “I can think of many ways to get out of a distressing situation.”). As a result of confirmatory factor analysis, fit index values were found to be (GFI = 0.96, AGFI = 0.92, RMR = 0.08, NNFI = 0.94, RFI = 0.90, CFI = 0.96, and RMSEA = 0.077). The internal consistency coefficient was 0.84, and the test-retest reliability coefficient was calculated as 0.81 for the agency thinking dimension, 0.78 for the thinking of alternative pathways dimension, and 0.86 for the scale’s total score. The internal consistency coefficients of the scale were found to be 0.73 for the thinking of alternative pathways sub-dimension, 0.76 for the agency thinking sub-dimension, and 0.84 for the total scale in this study.

### 3.3 Data analysis

It took students an average of 20 min to fill out the form. First, the forms were examined for incorrect coding (i.e., more than one answer to a question), and those with blank fields were excluded from evaluation. The data were examined for missing values, outliers, normality, and multicollinearity in the second stage. VIF and tolerance values were also examined for multicollinearity, and no multicollinearity was found between the variables. As a result of the examinations, 311 data points that met the required assumptions for analysis were analyzed. The data analysis used Pearson correlation analysis, serial mediation analysis, and the Bootstrap confidence intervals method to determine the relationships between variables. Given that the construct validity of the scales has been extensively established in prior research with similar populations, a new confirmatory factor analysis was not conducted. Instead, reliability for the current sample was confirmed through Cronbach’s alpha coefficients. Analysis was performed using SPSS 28 and SPSS AMOS 28. The serial mediation analysis performed with SPSS AMOS determines whether the mediation relationship is significant by using the highest and lowest values of the confidence intervals. The Bootstrap method does not include 0 (Hayes, 2017). In this context, the Bootstrap method with 5,000 samples was used, and the significance level of the mediation analysis was examined, considering the 95% confidence interval.

## 4 Results

### 4.1 Descriptive statistics and correlation results

Pearson Product-Moment Correlation was calculated to determine the relationships between trait EI, hope, career adaptability, and VOE. Correlation analysis and descriptive statistics results are given in Table 1.

**TABLE 1:** Correlation values between variables and descriptive statistics.

Variables	1	2	3	4
Vocational outcome expectations	–			
Hope	0.49**	–		
Trait emotional intelligence	0.33**	0.60**	–	
Career adaptability	0.56**	0.68**	0.48**	–
Mean	37.60	49.07	97.03	78.37
SD	4.85	6.94	15.30	8.27
Skewness	0.08	−0.30	0.00	−0.16
Kurtosis	0.33	0.07	−0.08	0.16

**p < 0.001.

As seen in Table 1, the skewness and kurtosis values of the variables of VOE, hope, trait EI, and career adaptability indicate a normal distribution (Büyüköztürk, 2015). A moderately significant positive correlation was found between the VOE and trait EI (*r* = 0.33), hope (*r* = 0.49), career adaptability (*r* = 0.56) variables (*p* < 0.01). A moderately significant positive correlation was found between hope and trait EI (*r* = 0.60), career adaptability (*r* = 0.68) variables (*p* < 0.01). A positive and moderately significant relationship was found between trait EI and career adaptability (*r* = 0.48) variables (*p* < 0.01).

### 4.2 Serial multiple mediational analyses

**FIGURE 1:**
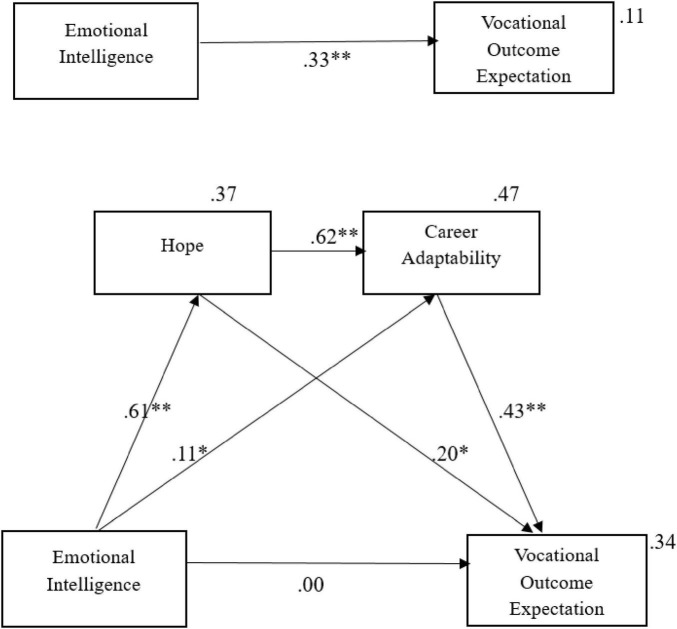
Serial multiple mediation of hope and career adaptability in the relationship between emotional intelligence and vocational outcome expectations. Values shown are standardized coefficients. *p < 0.05, **p < 0.001.

The serial mediation model was analyzed using IBM SPSS AMOS version 28. Specific indirect effects within the model were tested using Gaskin’s (2016) “Specific Indirect Effects Path and Serial Mediation” custom estimand. To evaluate the significance of the mediation relationships, 95% bootstrapped confidence intervals (CI) were utilized. The mediation is considered statistically significant (*p* < 0.05) if the upper and lower bounds of the confidence interval do not include zero. The results showed that the unstandardized total effect of trait emotional intelligence on vocational outcome expectation was significant [*B* = 0.105, SE = 0.019, 95% CI (0.069, 0.142)]. However, when the mediators were included in the analysis, the direct effect of trait emotional intelligence on vocational outcome expectation became statistically non-significant [*B* = 0.004, SE = 0.06, 95% CI (−0.128, 0.138)] (Figure 1).

Trait EI emerged as a positive predictor of hope (β = 0.61, *p* < 0.001) and career adaptability (β = 0.11, *p* < 0.05). Hope also showed positive effects on career adaptability (β = 0.62, *p* < 0.001) and VOE (β = 0.20, *p* < 0.05). In addition, career adaptability positively predicted VOE (β = 0.43, *p* < 0.001). The effect of trait EI on VOE was decomposed into one direct and three indirect pathways using unstandardized estimates: (1) direct effect of trait EI → VOE (B = 0.004); (2) indirect effect of trait EI → hope → VOE (B = 0.038); (3) indirect effect of trait EI → career adaptability → VOE (B = 0.014); (4) the combined indirect effect of trait EI → hope → career adaptability → VOE (B = 0.051) (Figure 1 and Table 2). Prior to the inclusion of the proposed mediators, trait EI was found to be a significant direct predictor of VOE. Nevertheless, the statistical significance of this direct path was completely attenuated once the mediating variables (hope and career adaptability) were integrated into the model. These findings suggest that hope and career adaptability fully mediate the relationship between trait EI and VOE.

**TABLE 2:** Serial multiple mediational analyses.

Parameter	Pathways	Effect value	BootSE	95% CI		*p* (two-tailed)
				BootLLCI	BootULCI	
c	tEI → VOE (total effect)	0.105	0.019	0.069	0.142	0.001
ab	Total indirect effect	0.104	0.014	0.077	0.133	0.001
a_1_b_1_	tEI → H → VOE	0.038	0.015	0.010	0.067	0.010
a_2_b_2_	tEI → CA → VOE	0.014	0.008	0.001	0.034	0.037
a_1_a_3_b_2_	tEI → H → CA → VOE	0.051	0.010	0.033	0.074	0.000

tEI, trait emotional intelligence; H, hope; CA, career adaptability; VOE, vocational outcome expectation. CI, confidence interval.

While the cross-sectional nature of our study limits causal inferences, the robustness of the proposed relational structure was evaluated by testing alternative structural models. In this context, we analyzed the hypothesized sequential mediation model (Model A) alongside the Full Reverse Causality Model (Model B) and the Mediator Order Reversal Model (Model C). Since all alternative structural models were saturated (*df* = *0*), the model fit indices (CFI, GFI, NFI) were obtained as 1, and the AIC and BIC values were non-discriminatory due to the equal number of parameters. This outcome is expected for saturated models and generally renders these criteria unsuitable for model comparison in this specific scenario. Consequently, the model selection was based on the strength of path coefficients, their significance levels, and theoretical consistency (see Table 3). The finding that the core relationship in Model B (CA → trait EI path) was statistically non-significant (*p* > 0.05) and the path coefficient in Model C (β = 0.51) was weaker and theoretically inconsistent compared to the corresponding path in Model A (β = 0.62) strongly supports the directional structure of trait EI → Hope → Career Adaptability → VOE. This evidence suggests that Model A provides the most robust empirical support for the theoretical linkages proposed and is therefore the preferred model.

**TABLE 3:** Testing alternative models.

Model name	Proposed relational chain	Critical path tested	Critical Path coefficient (β)	Critical path *p*-value	Decision and empirical rationale
Model A (hypothesized)	tEI →Hope →CA →VOE	Hope→ CA	0.62	< 0.01	Model selected (provides the strongest empirical relationship and is theoretically consistent)
Model B (full reverse causality)	VOE →CA →Hope → tEI	CA → tEI	0.12	> 0.05	Rejected (the critical path was found to be statistically non-significant)
Model C (mediator order reversal)	tEI → CA → Hope → VOE	CA → Hope	0.51	< 0.01	Rejected (provides weaker empirical support compared to the relationship in model A)

tEI, trait emotional intelligence; CA, career adaptability; VOE, vocational outcome expectations.

## 5 Conclusion and discussion

The findings of the study indicate that preservice teachers’ trait EI is significantly and positively associated with VOE. Furthermore, hope and career adaptability mediate the relationship between trait EI and VOE. When both hope and career adaptability are considered collectively, the results highlight the importance of emotional and motivational resources in the career development processes of preservice teachers. These findings enrich the literature on career psychology and teacher education by demonstrating the relationship between vocational outcome expectations and emotional and psychosocial factors.

The first hypothesis supported by the study is that trait EI is positively related to VOE. Upon reviewing the literature, no research was found that directly supports the findings of this study. However, indirect research supporting the result can be mentioned. Some studies have shown that trait EI and job satisfaction are related (Kafetsios and Zampetakis, 2008; San Lam and O’Higgins, 2012). In another study, Udayar et al. (2018) concluded that there is a positive and significant relationship between trait EI and perceived employability. Since VOE is an individual’s belief about whether their profession will provide them with the life they desire, this concept exhibits variability, along with the development of these positive traits. Trait EI is the capacity or skill to use emotions, regulate emotions, and solve problems to overcome a task or problem. Trait EI encompasses many competencies and traits that individuals need to perform the necessary actions to achieve positive outcomes related to their professions. Skills and competencies such as self-awareness and control of one’s own emotions, understanding the emotions of others, and socialization—all essential for teachers and preservice teachers who must constantly communicate with students and other education stakeholders—may enable them to have more positive expectations regarding their profession.

The second hypothesis supported by the study’s findings is that hope mediates the relationship between preservice teachers’ trait EI and VOE. Some studies in the literature support this research outcome (Batool et al., 2014; Fabio et al., 2018; Garcia et al., 2019; Mousa and Kamel, 2017; Sarıçam et al., 2015; Vela et al., 2015). Yılmaz (2019) found a negative relationship between students’ emotional intelligence and their levels of hopelessness. The competence regarding attitudes and values, one of the general competencies of the teaching profession, encompasses traits such as communication, collaboration, awareness, and a positive attitude (The Ministry of National Education [MoNE], 2017). These traits are closely related to trait EI. Having high trait EI may positively influence individuals’ awareness, knowledge, and skills for the teaching profession. Possessing the competencies required by the profession may contribute to an individual’s positive outlook on the future. Therefore, it may be positively related to preservice teachers’ VOE. Indeed, Garcia et al. (2019) found that hope is a significant predictor of vocational outcome experiences.

The third hypothesis supported by the study is that career adaptability mediates the relationship between preservice teachers’ trait EI and VOE. Individuals’ perceptions and attitudes influence VOE and are subject to continuous change (Işık, 2010). Trait EI is a valuable resource that enables individuals to cope with problems, solve them, and effectively regulate their emotions. In this context, individuals with high trait EI are likely to develop self-regulation skills, coping, and decision-making abilities concerning their careers. Career adaptability is a concept that enables individuals to cope with difficulties and influences their career perceptions in various ways (Savickas, 1997). When considered from this perspective, it is an expected result that individuals’ positive attitudes toward their careers are positively related to their VOE.

The fourth hypothesis supported by the study is that hope and career adaptability are independent and sequential mediators in the relationship between trait EI and VOE. Trait EI involves understanding and controlling emotions, problem-solving, regulating emotions, demonstrating competence in maintaining daily life, and feeling a driving force for successful performance. Teaching is a profession that requires many qualities. For example, Taşkaya (2012), in her study with preservice teachers, found that the phrase “good communication skills” was one of the most frequently mentioned characteristics that teachers should possess. Trait EI overlaps with many of the characteristics that teachers should possess, as previously mentioned. From this perspective, possessing the competencies required of a teacher may positively affect an individual’s level of hope. This study focused on two dimensions of hope: agency thinking and pathways thinking. Pathways thinking refers to the individual’s ability to create options to reach their goals, while agency thinking corresponds to the individual feeling strong and motivated in achieving their goals. These traits appear to overlap with career adaptability, which encompasses an individual’s ability to cope with problems, make informed decisions, and self-regulate in their professional life. Parallel to the study results, Büyükgöze-Kavas (2016) determined that hope is a significant predictor of career adaptability; Korkmaz and Cenkseven Önder (2019) determined that hope plays a partial mediating role in the relationship between life goals and career adaptability. The obtained results are similar to those reported in the study by Sarı et al. (2017). In that study, the researchers determined that self-transcendence, self-awareness, self-control, and self-management skills were significantly related to and important predictors of VOE. Self-control and self-management skills refer to a process in which the individual evaluates themselves and their purpose, and then self-regulates to achieve their goals. Self-control and self-management skills are similar in content to the dimensions of pathways thinking and agency thinking, which are components of hope. Agency thinking, which involves individuals’ thoughts about being able to make effective decisions to reach their goals, and the idea of pathways thinking, which involves the ability to generate new routes when faced with difficulties, can contribute to a positive expectation regarding vocational outcomes. Indeed, individuals’ abilities to create options for themselves and feel strong in achieving their goals may be related to the gains they derive from their profession and vocational values. Consequently, possessing positive traits and a positive outlook for daily life, the future, and career may positively influence an individual’s VOE.

### 5.1 Recommendations, limitations, and future directions

This research examines the effects of trait EI, hope, and career adaptability on preservice teachers’ VOE. The theoretical contributions can be summarized as follows: First, by highlighting the positive association of trait EI with VOE, the study provides theoretical support for the importance of trait EI in career guidance. Second, by demonstrating the mediating role of hope, the study reveals the importance of incorporating emotions into career guidance practices. Finally, the findings underscore the link between the development of skills such as competence awareness, self-regulation, and problem-solving, and positive VOE. These variables can effectively guide future interventions in teacher education to improve vocational outcomes. Regarding practical implications, the findings provide actionable insights for career counselors, educators, and university administrators. Since trait EI and hope significantly enhance vocational outcome expectations, teacher education programs should integrate psychoeducational interventions designed to cultivate these psychological resources. For instance, career counseling centers at universities can organize targeted workshops focusing on emotional regulation, goal-setting, and pathway thinking -key components of hope- to help preservice teachers build positive expectations about their future careers. Additionally, because career adaptability serves as a crucial mediator, curriculum developers could incorporate career construction training into the educational syllabus. By enhancing students’ career concern, control, curiosity, and confidence through applied counseling interventions, educators can better prepare preservice teachers to navigate the complex challenges of the teaching profession, ultimately fostering more positive vocational outcomes before they enter the workforce.

Despite these contributions, the study has several limitations that should be addressed in future research. First, the study’s cross-sectional design limits causal inference; therefore, future studies should employ longitudinal designs to examine causal relationships between these variables over time. Second, the reliance on self-report questionnaires may introduce potential method biases, such as social desirability bias. Third, the generalizability of the findings is limited, as the sample consists predominantly of female preservice teachers from only one country (Türkiye). Consequently, generalization to other populations or cultural contexts should be done cautiously, and cross-cultural studies are recommended to test the validity of the proposed model. Finally, while this study examines certain personal factors, social and cultural factors also influence expectations regarding a profession. In Türkiye, numerous economic, administrative, and social factors also affect the teaching profession. Because this study did not address these contextual factors, future empirical studies should thoroughly investigate environmental and contextual variables alongside personal factors.

## Data Availability

The datasets presented in this article are not readily available because the data obtained in our study have been restricted to the project team by the ethics committee in order to protect participant confidentiality. Therefore, making the data publicly available would not be consistent with ethical principles. The findings of our study are presented in detail within the article. Requests to access the datasets should be directed to hazelduru@uludag.edu.tr.
